# Robot-assisted anterior abdomino-vaginal mesh suspension for stress urinary incontinence associated with anterior compartment pelvic organ prolapse: technique, imaging workflow, and 12-month pilot outcomes

**DOI:** 10.3389/fsurg.2026.1825876

**Published:** 2026-05-26

**Authors:** Paolo Meloni, Sara Izzo, Giacomo Vigliercio, Terenzia Simari, Luciano Izzo, Claudia De Intinis, Silvia Lai, Nicole Asnong, Marcello Molle, Daniela Messineo, Paolo Izzo

**Affiliations:** 1 Obstetrics and Gynecology Unit, Imperia Hospital, ASL1 Imperiese, Imperia, Italy; 2 Plastic and Reconstructive Surgery Unit, Multidisciplinary Department of Medical-Surgical and Dental Specialties, Università Degli Studi Della Campania “L.Vanvitelli”, Naples, Italy; 3 Specialist Clinic for Gynecology and Obstetrics, ASL1 Imperiese, Imperia, Italy; 4 Ambulatorio Specialistico Ginecologia Ostetricia, ASL 1 Imperiese, Diano Marina, Italy; 5 "Pietro Valdoni” Department of Surgery, Policlinico “Umberto I”, “Sapienza” University of Rome, Rome, Italy; 6 Department of Translational and Precision Medicine, Nephrology Unit, Policlinico “Umberto I”, “Sapienza” University of Rome, Rome, Italy; 7 Department of Radiological, Oncological, and Anatomical Pathology Sciences, La Sapienza University, Rome, Italy

**Keywords:** anterior compartment, mesh suspension, MR defecography, pelvic floor MRI, pelvic organ prolapse, robotic surgery, stress urinary incontinence

## Abstract

**Background:**

Stress urinary incontinence (SUI) frequently coexists with pelvic organ prolapse (POP). Advanced prolapse may mask SUI, which can also develop *de novo* after prolapse reduction. Current management is based on standardized clinical evaluation using IUGA/ICS terminology and the POP-Q system, integrated with functional imaging such as transperineal ultrasound and dynamic pelvic floor MRI, including MR defecography when indicated. These tools allow accurate assessment of multicompartment prolapse and urethro-cervical mobility. This study describes a robot-assisted anterior abdomino-vaginal mesh suspension (AAVMS), revisiting a historical abdomino-vaginal mesh concept with modern robotic techniques.

**Materials and methods:**

From January 2024 to January 2025, sixteen women aged 55–65 years with anterior compartment POP and symptomatic SUI underwent robot-assisted AAVMS and were followed for 12 months. The primary endpoint was patient-reported resolution of SUI at 12 months. Secondary endpoints included perioperative outcomes, adverse events, POP-Q changes, and patient-reported quality of life (P-QOL) and sexual function (PISQ-12).

**Results:**

Operative time ranged from 50 to 90 min. Urinary catheter removal occurred on postoperative day 3, and discharge on days 8–10. At 6 months, 8/16 patients reported complete continence; this increased to 12/16 at 8 months and 15/16 at 12 months. One patient had persistent SUI. No intraoperative or postoperative complications or mesh-related adverse events were observed.

**Conclusions:**

Robot-assisted AAVMS was feasible and associated with progressive improvement in patient-reported continence over 12 months, with a favorable safety profile. Larger comparative studies with standardized objective endpoints are warranted.

## Background and Introduction

Pelvic organ prolapse (POP) and stress urinary incontinence (SUI) are frequently intertwined clinical entities. Anterior compartment prolapse may coexist with SUI, mask it through urethral kinking, or unmask it after prolapse reduction. Consequently, a modern surgical plan must integrate anatomical staging with a functional assessment of urethral support and mobility. It should also address the risk of persistent or *de novo* SUI following POP correction and incorporate patient preference regarding concomitant vs. staged anti-incontinence treatment.

Clinical evaluation has been standardized through the IUGA/ICS (1) terminology and the POP-Q staging system (2). Nevertheless, clinical staging alone may not fully capture multicompartment descent, levator ani integrity, or urethro-cervical mobility, particularly in cases of recurrent prolapse, discordance between symptoms and physical findings, or when minimally invasive reconstruction across multiple compartments is planned.

Functional imaging therefore complements POP-Q assessment (3). Translabial (4) or transperineal (5) ultrasound provides an accessible method for evaluating bladder neck descent, urethral rotation, levator hiatus dynamics, and levator ani muscle avulsions. Dynamic pelvic floor magnetic resonance imaging (MRI) (6) enables multiplanar visualization of all three pelvic compartments and supports standardized measurements, including the pubococcygeal line, H-line, and M-line. When defecatory symptoms or complex posterior compartment dysfunction are suspected, MR defecography adds a true evacuation phase and can demonstrate rectocele, enterocele or peritoneocele, internal intussusception, and pelvic floor dyssynergia, thereby facilitating a comprehensive, compartment-based reconstructive strategy.

From a surgical perspective, the minimally invasive era has consolidated robot-assisted pelvic reconstructive procedures. Robot-assisted sacrocolpopexy or sacrohysteropexy is widely regarded as the reference technique for apical defects (7), and Burch colposuspension (8) can also be reliably performed robotically in selected patients. Surgical options for SUI associated with POP include both abdominal and vaginal approaches, and the optimal strategy must be individualized. In patients with marked urethral hypermobility, Burch colposuspension remains a well-established option that can be performed via open, laparoscopic, or robotic access. Mid-urethral slings are widely used in contemporary practice; however, their role in the setting of POP repair must be considered in light of masking and unmasking phenomena, anatomical findings, and patient preference.

For apical prolapse, minimally invasive sacrocolpopexy (7) or sacrohysteropexy (8) has demonstrated durable anatomical correction and favorable functional outcomes and represents a cornerstone of modern pelvic reconstructive surgery. Alternative minimally invasive techniques, such as pectopexy (9) or lateral suspension, may be considered in selected clinical scenarios.

Against this background, we revisit a historical abdomino-vaginal mesh concept originally described via an open approach (10) and translate its core principles—broad vesicovaginal support, proximal abdominal anchorage, and complete peritoneal coverage—into a contemporary robot-assisted workflow. The AAVMS concept is designed to provide broad, tension-free support of the vesicovaginal junction and anterior fornix or vaginal cuff, addressing the functional consequences of anterior and apical descent as well as urethro-cervical mobility, particularly in patients whose anatomy suggests that an isolated anti-incontinence procedure may be insufficient. In this manuscript (reported in accordance with STROBE recommendations), we provide a detailed technical description of the procedure and report 12-month outcomes from a prospective pilot cohort.

## Material and methods

Sixteen women aged 55–65 years with symptomatic SUI in the setting of anterior compartment POP were enrolled between January 2024 and January 2025. The cohort was deliberately pragmatic and mixed, including patients with uterus *in situ* as well as post-hysterectomy patients, and including women undergoing AAVMS alone or in combination with concomitant robotic pelvic floor reconstruction when indicated. Given the pilot nature and small sample, no formal subgroup analyses were prespecified.

All patients underwent standardized clinical assessment including POP-Q staging and evaluation of urinary symptoms. Imaging was used to complement clinical staging and to guide reconstruction in complex or multi-compartment disease: TPUS and dynamic pelvic floor MRI were used to assess bladder neck descent, urethral mobility, levator hiatus dynamics, and compartmental prolapse. MR defecography was performed when posterior compartment symptoms or defecatory dysfunction suggested that an evacuation phase could modify the surgical plan. Urodynamic testing was performed according to contemporary indications (e.g., mixed symptoms, prior surgery, or clinical uncertainty).

Postoperative follow-up was scheduled prospectively at 1, 6, 8, and 12 months and conducted through standardized in-person clinical evaluations performed by the surgical team. At each visit, patients underwent a structured assessment that included symptom inquiry, a focused pelvic examination, and documentation of adverse events.

The primary endpoint was patient-reported resolution of stress urinary incontinence (SUI) at 12 months. Resolution was operationally defined *a priori* as (1) absence of stress-related urinary leakage during daily activities as reported by the patient, and (2) no pad use for stress incontinence. This endpoint was intentionally defined as patient-centered and symptom-based, consistent with the pilot cohort's exploratory feasibility objective.

Secondary endpoints included perioperative outcomes (operative time, catheterization duration, length of hospital stay), safety outcomes, anatomic outcomes based on POP-Q reassessment, and patient-reported outcome measures (PROMs), including the Prolapse Quality of Life questionnaire (P-QOL) and the Pelvic Organ Prolapse/Urinary Incontinence Sexual Questionnaire-12 (PISQ-12).

All perioperative and postoperative adverse events were prospectively recorded from the day of surgery through the 12-month follow-up visit. Complications were graded using the Clavien-Dindo classification, and mesh-specific events were systematically screened for at each in-person evaluation.

## Surgical procedure

In our current protocol, robot-assisted AAVMS is indicated for women with symptomatic stress urinary incontinence (SUI) associated with anterior compartment pelvic organ prolapse (POP), in whom clinical and imaging evaluation demonstrates significant urethro-cervical mobility and vesicovaginal descent. The procedure may be performed either as a standalone anterior support and anti-incontinence intervention or in combination with concomitant robotic apical and/or posterior compartment repairs in cases of multi-compartment prolapse, reflecting real-world clinical practice. Contraindications include active pelvic infection, untreated malignancy, inability to comply with postoperative follow-up, and clinical conditions associated with an unacceptably high risk of mesh-related complications. Given the regulatory and clinical sensitivity surrounding the use of mesh, careful patient selection and comprehensive informed consent are integral parts of the protocol.

A macroporous monofilament polypropylene mesh strip, approximately 4 cm in width and of sufficient length to allow tension-free cranial anchorage, is prepared. Nonabsorbable sutures are used for fixation to robust fascial structures, while vesicovaginal fixation is performed using either slowly absorbable or nonabsorbable sutures according to tissue quality and surgeon preference. In all cases, complete peritonealization is planned to minimize mesh contact with intra-abdominal viscera.

Patients are positioned in dorsal lithotomy with steep Trendelenburg. Following bladder catheterization, a standard robotic pelvic port configuration is established. The peritoneum over the anterior compartment is incised, and the vesicovaginal plane is carefully developed to the level of the anterior vaginal fornix or, in post-hysterectomy patients, to the vaginal cuff. Meticulous hemostasis is maintained throughout the dissection, with continuous attention to preserving bladder integrity.

The mesh strip is positioned to straddle the vesicovaginal anterior fornix. Distal fixation is achieved with interrupted bilateral sutures incorporating deep vesicovaginal tissues and the pubocervical fascia; additional fixation points may include the cervix or uterine isthmus, or the vaginal cuff when appropriate. The aim is to provide supportive contact without tissue constriction. The proximal segment of the mesh is then routed cranially through a developed anterior abdominal wall plane, ensuring a tension-free course, and anchored to the posterior rectus sheath and/or abdominal aponeurosis using nonabsorbable sutures placed over an adequate length to prevent slippage while avoiding excessive elevation. When required, transfascial suturing is employed to obtain secure fixation to the abdominal wall fascia.

Finally, a broad peritoneal flap is mobilized and closed over the entire mesh to ensure complete coverage and eliminate exposed edges. When multi-compartment defects are present, the procedure may be combined with robot-assisted apical suspension, such as sacrocolpopexy or sacrohysteropexy, and/or posterior compartment repair. The sequence of procedures is individualized according to anatomical findings; however, complete peritonealization of all mesh components remains mandatory.

The procedure should be tension-free. The goal is durable support rather than aggressive elevation. Mesh fixation must be deep and symmetric at the vesicovaginal junction, while cranial anchorage should be secure but not taut. In post-hysterectomy patients, careful adhesiolysis and clear definition of the vaginal cuff are critical. Because this is not a guideline-codified technique, meticulous documentation, targeted informed consent, and structured follow-up are essential.

## Results

The mean age of the cohort was 60.8 ± 3.1 years, with a mean BMI of 26.9 ± 3.4 kg/m^2^. All patients were postmenopausal. Seven patients had a history of previous pelvic surgery, including hysterectomy in four cases and prior prolapse repair in tre. AAVMS was performed as a standalone procedure in 10 patients, while 6 patients underwent concomitant pelvic floor reconstruction (sacrocolpopexy or sacrohysteropexy, and/or posterior compartment repair),

Operative time ranged from 50 to 90 min. All patients were mobilized on postoperative day 1. The urinary catheter was removed on postoperative day 3, and patients were discharged between postoperative days 8 and 10. At 6 months, 8 of 16 women (50.0%; 95% CI 24.7–75.3) reported complete continence and subjective well-being, while the remaining 8 reported residual stress urinary leakage. At 8 months, 12 of 16 women (75.0%; 95% CI 47.6–92.7) were continent and well, and 4 reported residual mild leakage. At 12 months, continence and well-being were reported by 15 of 16 women (93.8%; 95% CI 69.8–99.8). One patient (6.2%; 95% CI 0.2–30.2) reported persistent stress urinary incontinence and was classified as a treatment failure (Table 1). No intraoperative complications were recorded. Specifically, there were no bladder, ureteral, bowel, or vascular injuries, and no procedures required conversion to open surgery. Postoperatively, no complications of Clavien–Dindo grade II or higher were observed (Table 2). In addition, no Clavien–Dindo grade I events requiring pharmacological or procedural intervention were documented during either the index hospitalization or the follow-up period. At 12 months, no mesh-related adverse events were identified. There were no cases of vaginal mesh exposure or erosion on clinical examination, no reports of *de novo* chronic pelvic pain attributable to the mesh, no mesh infections, and no reoperations related to the index procedure. Quality-of-life outcomes, assessed using the P-QOL questionnaire, showed improvement by 6 months and remained stable thereafter (Table 3). Patient-reported sexual function, evaluated with the PISQ-12, improved in most participants. Qualitative reporting suggested reductions in dyspareunia and prolapse-related embarrassment in approximately 90% of women. Formal objective measures and subgroup analyses were not prespecified in this pilot cohort.

**Table 1:** Continence outcomes (*n* = 16).

Time point	Continent/well	Residual SUI	Notes
6 months	8/16 (50.0%; 95% CI 24.7–75.3)	8/16	Progressive improvement expected
8 months	12/16 (75.0%; 95% CI 47.6–92.7)	4/16	Residual cases improved over time
12 months	15/16 (93.8%; 95% CI 69.8–99.8)	1/16 (6.2%; 95% CI 0.2–30.2)	One treatment failure (persistent SUI)

**Table 2:** Safety outcomes (*n* = 16).

Safety endpoint (12 months)	n/N (%)
Intraoperative adverse events	0/16 (0%)
Conversion to open surgery	0/16 (0%)
Postoperative complications (Clavien–Dindo ≥ II)	0/16 (0%)
Reoperation	0/16 (0%)
Mesh-related adverse events (exposure/erosion/infection)	0/16 (0%)

**Table 3:** Patient-reported outcomes (P-QOL and PISQ-12): qualitative summary.

Time point	P-QOL (POP-related QoL)	PISQ-12/sexual function (qualitative summary)
Baseline	Baseline assessment	Baseline assessment
6 months	Improvement in symptom and activity domains	≈90% reported reduction of dyspareunia/embarrassment; gradual resumption of sexual activity
8 months	Stabilization of improvement	Sustained improvement trend
12 months	Maintenance of improvement	High overall satisfaction trend; formal subgroup analysis not prespecified

## Discussion and conclusion

The abdomino-vaginal mesh concept was originally described using an open abdominal approach (11). Its key steps included development of the vesicovaginal plane at the level of the anterior fornix, followed by placement of a polypropylene mesh strip approximately 4 cm in width across this region. The mesh was secured with deep bilateral fixation to the vesicovaginal tissues, while an 8–10 cm proximal segment was anchored to the posterior surface of the rectus muscles and the corresponding aponeurotic plane. The procedure concluded with complete peritoneal coverage. The underlying rationale was to achieve a tension-free configuration, thereby minimising chronic traction on pelvic tissues.

Our robot-assisted adaptation preserves these fundamental principles while taking advantage of enhanced visualisation and increased precision in suturing. This approach facilitates meticulous dissection and secure mesh fixation within a minimally invasive setting. The rationale is grounded in pelvic floor functional anatomy: by supporting the vesicovaginal junction and restoring a stable relationship among the anterior vaginal wall, the bladder base, and the proximal urethra, the procedure aims to mitigate stress urinary leakage related to urethral hypermobility in the setting of anterior and/or apical descent (12, 13). The robotic platform facilitates precise dissection of the vesicovaginal plane, controlled and symmetric mesh fixation to deep tissues, and reliable complete peritoneal coverage. The emphasis placed on functional imaging is integral rather than ancillary. In real-world mixed cohorts, surgical planning is often influenced by interactions among pelvic compartments that may not be fully captured by clinical staging alone (14). Dynamic pelvic floor MRI and, when indicated, MR defecography (15) can document multicompartment descent, levator hiatus dynamics, and associated posterior compartment abnormalities. The inclusion of representative preoperative and postoperative imaging is intended to make the anatomical–functional rationale explicit, promote standardized reporting, and support multidisciplinary communication among urogynecology, urology, and radiology (16).

In this pilot cohort, patient-reported continence outcomes improved progressively over time, with 15 of 16 women reporting continence and well-being at 12 months and one persistent failure. The absence of intraoperative complications and mesh-related adverse events is reassuring but must be interpreted cautiously given the small sample size and limited follow-up. The single failure highlights that no anti-incontinence strategy is universally effective and suggests that factors such as intrinsic sphincter deficiency, detrusor overactivity, or other unrecognized functional contributors may limit the efficacy of a purely support-based approach in selected patients. This observation reinforces the importance of careful patient selection and, when clinically indicated, preoperative urodynamic evaluation. Several limitations must be acknowledged. The study is limited by its small sample size, noncomparative design, and heterogeneity resulting from concomitant reconstructive procedures. Outcomes were predominantly patient-reported, and standardized objective continence testing and systematic postoperative imaging were not prespecified for the entire cohort. These factors preclude causal inference and limit the generalizability of the findings.

Future investigations should incorporate standardized objective continence endpoints, such as cough stress testing at a predefined bladder volume, pad testing, or validated continence questionnaires with prespecified thresholds. Systematic complication reporting using Clavien–Dindo classification and mesh-specific definitions, predefined imaging endpoints on dynamic MRI or MR defecography, and comparative study designs against established minimally invasive strategies—such as robotic Burch colposuspension, mid-urethral sling18 procedures, or prophylactic anti-incontinence approaches performed at the time of apical suspension—will be essential to define the role of this technique.

In summary, in this prospective pilot cohort of 16 women undergoing robot-assisted AAVMS for stress urinary incontinence associated with anterior compartment prolapse, patient-reported continence improved over 12 months, with one treatment failure and no recorded complications. The technique appears feasible and anatomically motivated; however, its relative effectiveness and safety compared with established minimally invasive anti-incontinence procedures require validation in larger, comparative studies with standardized clinical and imaging outcomes. Given the limited sample size and follow-up duration, the present findings should be interpreted as preliminary safety and feasibility data. Ongoing surveillance beyond 12 months is underway to monitor for delayed mesh-related events.

## Data Availability

The original contributions presented in the study are included in the article/Supplementary Material, further inquiries can be directed to the corresponding author.
